# The Osteosarcoma Tumor Microenvironment: Supportive of Tumor Progression, Hostile to Immunity, but Therapeutically Targetable

**DOI:** 10.3390/cancers18193239

**Published:** 2026-10-08

**Authors:** Lidia Tarone, Irene Fiore Merighi, Elisa Tirtei, Davide Giacobino, Emanuela Maria Morello, Federica Cavallo, Federica Riccardo

**Affiliations:** 1 Laboratory of OncoImmunology, Molecular Biotechnology Center “Guido Tarone”, Department of Molecular Biotechnology and Health Sciences, University of Turin, 10126 Turin, Italy; lidia.tarone@unito.it (L.T.); irene.merighi@unito.it (I.F.M.); federica.riccardo@unito.it (F.R.); 2 Pediatric Onco-Hematology Department, Regina Margherita Children’s Hospital, 10126 Turin, Italy; elisa.tirtei@unito.it; 3 Department of Public Health and Pediatrics, University of Turin, 10124 Turin, Italy; 4 Department of Veterinary Sciences, University of Turin, 10095 Grugliasco, Italy; davide.giacobino@unito.it (D.G.); emanuela.morello@unito.it (E.M.M.)

**Keywords:** osteosarcoma, B7-H3, CSPG4, tumor microenvironment modulation

## Abstract

Osteosarcoma (OSA) remains the most common primary malignant bone tumor of children and young adults, with limited survival improvements in patients with relapsed or metastatic disease despite multimodal chemotherapeutic treatment. Beyond the intrinsic heterogeneity and malignancy of tumor cells, OSA progression and therapeutic resistance are strongly influenced by the specialized bone tumor microenvironment (TME). Interactions among tumor, immune and stromal cells generate a complex ecosystem characterized by immune suppression, hypoxia and metabolic adaptation, representing a major challenge for effective (immune) therapeutic approaches. In this review, we discuss how the OSA microenvironment shapes tumor evolution and therapeutic response, with particular emphasis on the mechanisms that limit effective immune-mediated tumor control. We further examine the rationale for combining tumor-directed and microenvironment-directed immunotherapies. Within this context, B7-H3/CD276 represents a relevant example of a multifunctional molecule at the interface between tumor biology and immune regulation. B7-H3 is associated with aggressive tumor features and therapy resistance, while its immunoregulatory activities contribute to the establishment of an immune-restrictive TME. Consequently, B7-H3-targeted therapies may exert complementary mechanisms of action, either by directly eliminating B7-H3-expressing tumor cells or by modulating the TME to overcome immunosuppressive barriers. This dual activity provides rationale for combination strategies integrating B7-H3-targeting with antigen-directed immunotherapies, including those targeting the novel OSA-associated antigen, chondroitin sulfate proteoglycan 4, where effective antitumor immunity may require simultaneous modulation of the local immune context. We then discuss how advanced preclinical models, comparative oncology, and emerging biomarker-guided approaches may accelerate the clinical translation of more effective immunotherapeutic strategies for OSA.

## Simple Summary

Osteosarcoma is the most common malignant bone tumor in children and adolescents. Although surgery and chemotherapy have improved the outcome of many patients, effective options remain limited for those with recurrent or metastatic disease. This restricted progress is not explained only by the biology of tumor cells but also by the surrounding tumor microenvironment, which protects cancer cells and limits the immune response. The aim of this review is to depict the osteosarcoma microenvironment and discuss how it influences disease evolution and treatment resistance, with particular attention to B7-H3, a molecule involved in tumor aggressiveness and immune regulation. We discuss the potential of B7-H3-targeted approaches to reshape the tumor microenvironment and their integration with antigen-directed immunotherapies, including those targeting the chondroitin sulfate proteoglycan 4. Finally, we highlight the relevance of improved preclinical models, including comparative oncology, to support the development of more effective and personalized treatments for osteosarcoma patients.

## 1. Introduction: Osteosarcoma as a Therapeutic and Microenvironmental Challenge

Osteosarcoma (OSA) is the most common primary malignant bone tumor of childhood, adolescence, and young adulthood [1,2]. Despite the multi-modal treatment approach with surgery and neo-adjuvant and adjuvant poli-chemotherapy, survival has plateaued for more than 40 years [3,4,5]. Approximately 40% of patients relapse, and metastatic disease remains the leading cause of death, with 5-year survival rates still around 20% [1,6]. Moreover, treatment-related toxicities significantly compromise long-term quality of life [1]. The modest progress in OSA therapy contrasts sharply with advances achieved in other solid tumors. One reason is the absence of recurrent, easily druggable oncogenic drivers [6,7]. Another key limitation is the profound OSA genetic instability and interpatient heterogeneity, which complicate biomarker-driven therapy [8].

An increasingly important focus in the development of novel effective OSA-targeting therapies is the recognition that OSA is not only a disease of malignant osteoid-producing cells, but also it is characterized by a specialized bone tumor ecosystem, which limits the efficacy of targeted therapies [9]. Tumor cells evolve within a mineralized, hypoxic, mechanically stiff, immune-suppressive, and spatially heterogeneous microenvironment that actively shapes tumor progression, metastatic dissemination, and therapeutic resistance [10].

OSA should not be considered an immunologically inert tumor. Indeed, the use of mifamurtide, an immunomodulator that activates monocytes and macrophages, outlines that immune manipulation can be clinically meaningful when aligned with OSA biology [11,12,13]. However, even if immune checkpoint inhibitors (ICI) have transformed the treatment of many solid cancers, they have shown limited activity in OSA as monotherapy (objective response rate of approximately 5%) [14,15]. This evidence suggests that canonical PD-1/PD-L1 axis inhibition may be insufficient when the tumor bed lacks effective T-cell infiltration, contains abundant suppressive myeloid cells, and imposes physical and metabolic barriers to immune function, as is the case in OSA.

The central challenge is shifting from whether OSA can be targeted immunologically to how its uniquely restrictive tumor microenvironment (TME) can be overcome and reprogrammed.

Within this context, B7-H3, also known as CD276, has emerged as a particularly attractive molecule to be investigated. B7-H3 is frequently overexpressed in malignant tissues, including OSA, while showing comparatively restricted or lower protein expression in many normal tissues [16,17]. It has been implicated in immune suppression, tumor invasion, angiogenesis, metabolic adaptation, metastatic behavior, and therapy resistance [18]. Importantly, it is also a highly accessible cell-surface antigen, making it suitable for antibody-based therapies, antibody–drug conjugates (ADC), and chimeric antigen receptor (CAR)-T-cell approaches [19]. Therefore, it should be considered not only as a potential immune checkpoint-like molecule, but more broadly as a multifunctional antigen with immunoregulatory activities and therapeutically targetable.

Furthermore, our previous work on chondroitin sulfate proteoglycan 4 (CSPG4) [20,21] provides an additional perspective on antigen-directed immunotherapy in OSA. CSPG4 has been investigated as an OSA-associated antigen with functional relevance in tumor biology and as a potential target for vaccine-based approaches [22,23]. While CSPG4 vaccination elicited effective antitumor immune responses, subsequent analyses revealed the emergence of B7-H3 upregulation as a potential immune escape mechanism that may contribute to limiting vaccine efficacy.

The scope of the present review is distinct from that of our recent review published in *Cancers*, which examined CSPG4 together with xCT and Toll-like receptor 2 (TLR2) as complementary molecular vulnerabilities involved in major cancer hallmarks in OSA [24]. Here, the focus is instead on the OSA TME and on how its cellular, structural, metabolic, and immune components influence tumor progression, immune restriction, and therapeutic response. B7-H3 is therefore considered within this microenvironmental framework, while CSPG4-directed immunotherapy provides an example of how the efficacy of tumor-antigen targeting may depend on the permissiveness of the surrounding TME.

In particular, here we discuss how OSA TME contributes to immune escape, metastatic progression, and therapeutic resistance, with a particular focus on B7-H3 as an immunoregulatory molecule and therapeutic target. We further discuss how insights gained from CSPG4-directed vaccination might serve as an example to support the development of combinatorial immunotherapeutic strategies aimed at integrating tumor-specific immune activation with microenvironmental reprogramming and highlight the major unresolved questions that need to be addressed to translate these concepts into effective therapeutic strategies.

## 2. The Tumor Microenvironment in Osteosarcoma: Supportive for Tumor Growth, Hostile to Immunity

TME is not a passive scaffold surrounding malignant cells. It is a dynamic ecosystem in which tumor cells interact with the surrounding cellular and non-cellular components and can exert either restraining or promoting effects on tumor development, depending on the stage of disease and the balance of local interactions. In early phases, components of the microenvironment may contribute to tissue homeostasis and immune surveillance, limiting aberrant cancer cell expansion. However, during tumor progression, this equilibrium is frequently disrupted, and the TME is progressively reprogrammed toward a state that is supportive for the tumor but hostile to antitumor immunity and drug penetration.

**Figure 1:**
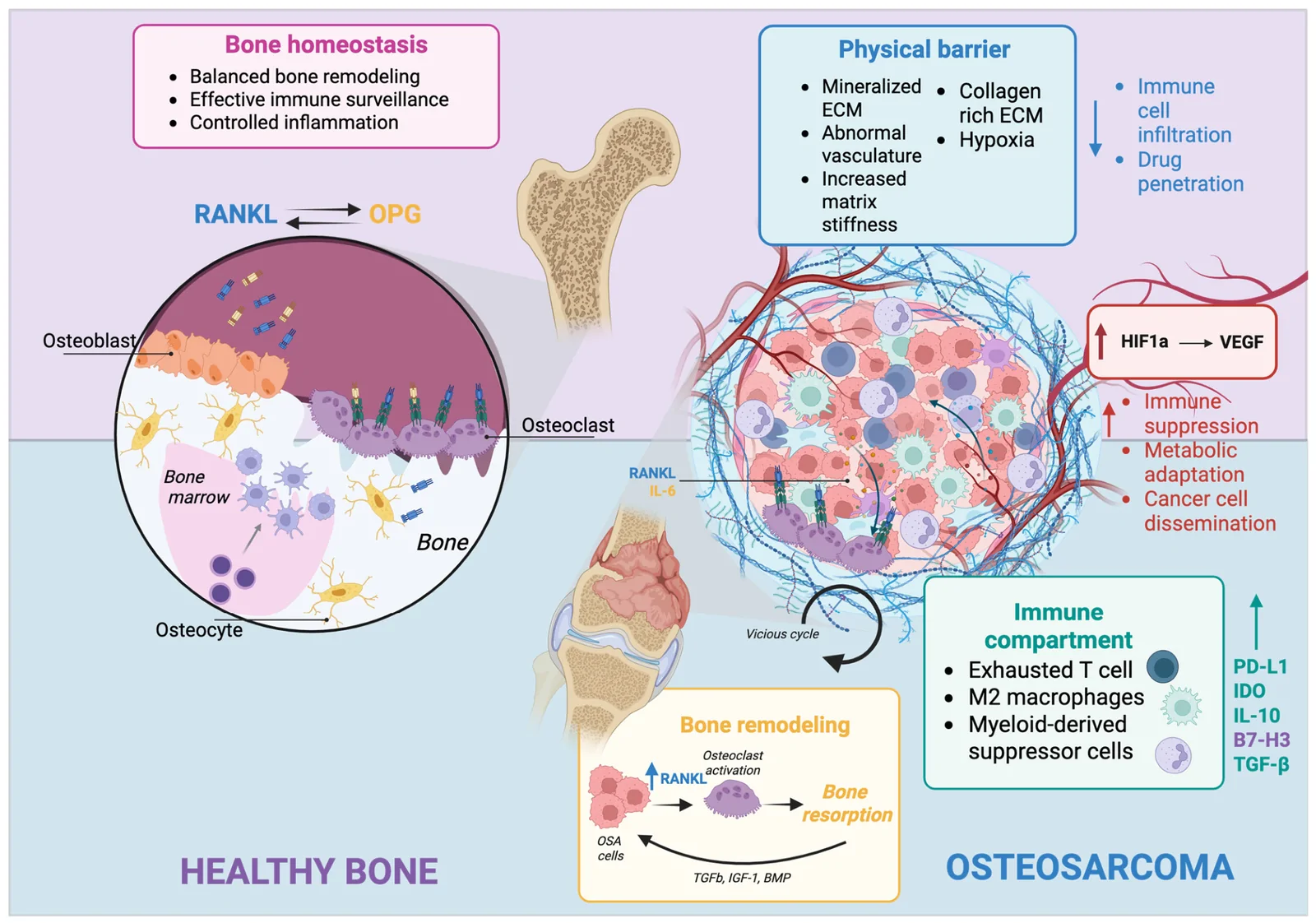
**The osteosarcoma tumor microenvironment as a dual ecosystem.** In healthy bone, coordinated interactions among osteoblasts, osteoclasts, osteocytes, and bone marrow components—including immune cells—maintain balanced bone remodeling, effective immune surveillance, and controlled inflammation, with RANKL/OPG signaling contributing to the regulation of osteoclastogenesis. In OSA, tumor cells disrupt this regulation, and it is established a TME supportive for tumor growth and metastasis, but hostile to immune infiltration and therapy. The schematic illustration shows the major cellular and non-cellular components of the OSA niche, including tumor cells, tumor-associated macrophages, myeloid-derived suppressor cells, T cells, mesenchymal stromal cells, endothelial cells, osteoblasts, osteoclasts, and the mineralized and collagen-rich extracellular matrix. Their interactions contribute to osteoclast activation, extracellular matrix remodeling, angiogenesis, hypoxia, metabolic adaptation, and the accumulation of immunosuppressive mediators. Lines ending with a dot indicate the molecules or cells represented at the corresponding location; upward arrows indicate an increase and downward arrows indicate a reduction. Created with BioRender (https://BioRender.com/pqg2gu0), accessed on 28 September 2026.

This duality is central to OSA biology, where the highly complex TME is composed of both cellular and non-cellular components that continuously interact to influence disease progression and therapeutic outcome (Figure 1). Cellular elements include malignant cells, myeloid cells, mesenchymal stromal cells, endothelial cells, osteoclasts, immune populations, and other cellular compartments [25]. In parallel, non-cellular components such as the extracellular matrix (ECM) and soluble mediators, like cytokines, chemokines, metabolites and extracellular vesicles play fundamental roles in shaping tissue architecture and intercellular communication.

Within this specialized environment, the interplay between cellular and acellular elements generates a unique biological context (Figure 1). The mineralized matrix, collagen-rich extracellular scaffold, abnormal vasculature, hypoxic gradients, osteolytic/osteoblastic remodeling, and increased interstitial pressure collectively create a physical and biochemical barrier to immune-cell entry and therapeutic delivery. At the same time, these same features provide signals that sustain tumor-cell plasticity, metabolic adaptation, survival under stress, and metastatic competence.

Thus, the OSA microenvironment simultaneously supports tumor progression while restricting effective antitumor immunity, making it both a driver of disease evolution and a major determinant of therapeutic resistance.

### 2.1. Immune Restriction and Myeloid Dominance

The immune compartment of OSA is frequently described as “cold”, “immune-excluded,” or “non-T-cell-inflamed” [26,27]. However, this does not mean that immune cells are absent. Rather, immune infiltration is often skewed toward suppressive or dysfunctional states. In this context, the failure of antitumor immunity may depend not only on the number of immune cells present within the lesion, but also on their localization, activation state, and ability to interact productively with malignant cells.

Tumor-associated macrophages (TAMs), especially M2-like or alternatively activated states, and myeloid-derived suppressor cells (MDSCs) are prominent in many OSA lesions. Their accumulation is not random, but reflects an active process driven by tumor- and stroma-derived signaling networks that shape immune cell recruitment and functional reprogramming. These populations can inhibit T-cell priming and effector function, promote angiogenesis, remodel the ECM, and support metastatic dissemination. Recent studies and reviews specifically emphasize myeloid dominance, macrophage heterogeneity, immune exclusion, and spatial complexity as defining features of the OSA immune microenvironment [28,29].

The functional plasticity of TAMs also makes them attractive therapeutic targets in OSA. In particular, strategies aimed at repolarizing tumor-supportive macrophages toward pro-inflammatory phenotypes are being explored [30]. Among these, M1 macrophage-derived vesicles have emerged as potential immune-modulatory drug-delivery platforms. After initial proof-of-concept studies in other tumor models [31], cisplatin-loaded M1 macrophage-derived vesicles were recently evaluated in OSA, where they reduced tumor burden and systemic toxicity while increasing the intratumoral M1/M2 macrophage ratio [32]. These findings suggest that macrophage-directed vesicle strategies may simultaneously improve drug delivery and partially reprogram the OSA immune microenvironment.

Among the molecular pathways linking tumor cells to suppressive myeloid remodeling, the CSF1/CSF1R axis is particularly relevant. CSF1R signaling regulates the survival, differentiation, recruitment, and functional polarization of monocytes, macrophages, and osteoclast-lineage cells. In OSA models, tumor-cell-derived CSF1 has been implicated in macrophage recruitment to the TME, while CSF1R expression has been described in both myeloid and tumor-cell compartments [33,34]. Therefore, CSF1/CSF1R signaling may contribute to OSA progression through complementary mechanisms, including the maintenance of macrophage-rich immune niches, the support of osteoclast-lineage biology, and, in some contexts, direct effects on CSF1R-positive tumor-cell subsets.

Nevertheless, the role of macrophages and CSF1R-related signatures in OSA should be interpreted carefully. Macrophage states are heterogeneous and cannot be reduced to a simple M1/M2 dichotomy. Depending on spatial localization, activation state, and disease context, macrophage-rich areas may reflect either antitumor inflammation or tumor-supportive immune remodeling. Collectively, current evidence supports a model in which immune dysfunction in OSA reflects not simply reduced immune-cell abundance, but the spatial and functional organization of suppressive myeloid populations and their interactions with tumor and stromal compartments. However, it remains unclear which myeloid states and spatial configurations are most consistently associated with therapeutic resistance, and whether these features are stable across primary, recurrent, and metastatic disease.

### 2.2. Bone Remodeling, Metabolic Stress and Physical Barriers

The bone niche adds another layer of specificity and complexity to the OSA microenvironment. Osteoblasts, osteoclasts, mesenchymal stem cells (MSC), mineralized matrix, and bone-remodeling pathways are not bystanders. OSA cells can engage osteoblast–osteoclast signaling loops, including RANKL–RANK-mediated osteoclast activation, creating a “vicious cycle” of bone destruction, growth-factor release, and tumor progression [35]. This is particularly relevant when exploiting in vitro systems because osteoclasts and osteoblast-lineage cells are absent or poorly represented, though they may strongly influence tumor invasion, immune-cell positioning, and therapeutic response.

Additional key features of the OSA niche, such as hypoxia, vascular dysfunction, metabolic stress, ECM remodeling, and increased mechanical stiffness, further contribute to immune restriction. Hypoxia develops because of rapid tumor growth, disorganized vasculature, and dense matrix architecture. Stabilization of hypoxia-inducible pathways, particularly HIF-1α-dependent signaling, promotes angiogenic programs, including VEGF-driven vascular remodeling, and supports tumor-cell adaptation to oxygen and nutrient deprivation. However, hypoxia also has important immunological consequences: it can impair cytotoxic T-cell and natural killer (NK)-cell activity, favor the accumulation and suppressive function of myeloid populations, and promote the release of mediators that reinforce immune escape [36,37].

Hypoxia is closely linked to metabolic reprogramming. OSA cells exposed to oxygen and nutrient limitation can shift toward glycolytic metabolism, leading to lactate accumulation and extracellular acidification. These metabolic changes create a hostile environment for effector lymphocytes, whose proliferation and cytotoxic function are highly sensitive to nutrient availability, pH, and oxidative stress. In parallel, lactate-rich and hypoxic niches may favor tumor-supportive macrophage states and contribute to the persistence of MDSCs and regulatory T cells. Thus, the metabolic organization of the OSA microenvironment may help explain why immune cells can be present within tumor lesions but fail to mount an effective antitumor response. Interestingly, metabolic rewiring in OSA is not restricted to tumor cells but also extends to the surrounding stromal compartment. The classical Warburg effect describes the ability of cancer cells to preferentially utilize aerobic glycolysis, converting glucose into lactate despite the presence of oxygen, as an adaptation process [38,39,40]. This metabolic adaptation produces ATP rapidly, that can support biosynthetic pathways required for proliferation, although at the cost of reduced energetic efficiency compared with oxidative phosphorylation. However, in OSA, evidence suggests that metabolic adaptation may also rely on a bidirectional coupling with MSCs. OSA cells can release soluble factors that “educate” neighboring MSCs, inducing a glycolytic phenotype characterized by enhanced glucose uptake, lactate production, pyruvate release, and broader metabolic remodeling. These reprogrammed MSCs acquire a tumor-supportive phenotype and function as metabolic suppliers within the tumor niche [41,42]. Consequently, OSA cells can exploit and import these stromal-derived metabolites, utilizing them as an oxidative substrate through mitochondrial oxidative phosphorylation. This metabolic cooperation, referred to as the reverse Warburg effect, provides several advantages to tumor cells by preserving glucose availability, maintaining mitochondrial activity, and increasing adaptability to hypoxia and nutrient stress. Rather than representing an isolated metabolic adaptation of malignant cells, this process highlights the OSA microenvironment as a coordinated metabolic ecosystem in which tumor and stromal cells cooperate to sustain tumor progression and resistance.

The physical properties of the OSA matrix provide an additional barrier. The osteoid-rich and collagen-dense ECM can limit immune-cell migration and drug diffusion, while also transmitting mechanical signals to tumor and stromal cells. Increased stiffness activates pathways, including integrin/FAK signaling, Rho/ROCK-dependent cytoskeletal remodeling, YAP/TAZ activation, and matrix-sensing receptors such as discoidin domain receptors. These pathways can promote tumor-cell proliferation, motility, invasion, stem-like features, and therapy resistance. Moreover, ECM remodeling and matrix stiffness may indirectly influence immunity by altering chemokine gradients, endothelial permeability, macrophage polarization, and the spatial distribution of T cells within the tumor bed [43,44,45].

Together, these stromal, immune, metabolic and physical features explain why OSA lesions may contain immune cells unable to generate effective antitumor immunity. Rather than being simply immune-devoid, OSA should be viewed as a spatially organized and mechanically constrained tumor ecosystem in which immune-cell recruitment, localization, and function are shaped by the bone niche [26]. This interpretation is particularly relevant when considering immune-targeted strategies, including B7-H3 targeting and antigen-directed approaches such as CSPG4-directed immunotherapy, whose efficacy may depend on both antigen expression and microenvironmental permissiveness.

An important unresolved issue is whether these physical and metabolic barriers represent stable properties of OSA lesions or dynamically evolve during chemotherapy and immunotherapy, and whether they can be therapeutically modified without compromising tissue homeostasis.

## 3. B7-H3: Beyond the Classical Immune-Checkpoint Paradigm

B7-H3 belongs to the B7 protein family and the B7/CD28 immunoregulatory superfamily. It is a type I transmembrane glycoprotein with immunoglobulin-like extracellular domains. In humans, two major isoforms have been described: 2Ig-B7-H3 and 4Ig-B7-H3. The 2Ig-B7-H3 isoform contains one IgV-like and one IgC-like domain, whereas the 4Ig-B7-H3 contains a tandem duplication of this IgV-IgC unit, resulting in two highly homologous IgV-IgC pairs. The 4Ig-B7-H3 isoform is the predominant form in humans. By contrast, murine B7-H3 contains a single IgV-IgC pair and corresponds to the 2Ig-B7-H3 form [46]. Comparative genomic analyses indicate that the 4Ig-B7-H3 isoform is also present in several non-primate mammals, including dogs, although the functional relevance of species-specific isoform usage remains incompletely defined [47,48].

B7-H3 biology remains unusually complex because, unlike PD-L1 or CTLA-4 ligands, its definitive receptor(s) and signaling partners have not been established yet. TLT-2 has been proposed in some experimental systems, but this interaction has not been consistently validated across human immune contexts. Structural and mechanistic studies, therefore, continue to describe B7-H3 as an atypical B7 family member without a universally accepted physiological cognate receptor [49,50].

Under physiological conditions, B7-H3 mRNA is detectable across a broad range of normal human tissues, whereas B7-H3 protein expression is comparatively low, suggesting tightly post-transcriptional and post-translational control mechanisms, including microRNA regulation [19,51]. Contrarily, B7-H3 protein is frequently upregulated in tumor cells, tumor-associated vasculature, stromal cells, and immune cells [19]. In tumors, it exerts both immune and non-immune pro-tumorigenic functions, including suppression of T-cell activation, modulation of innate immune activity, promotion of migration and invasion, angiogenesis, chemoresistance, and metabolic reprogramming [52].

### 3.1. Immune-Mediated Functions of B7-H3

The immune effects of B7-H3 are context-dependent and remain incompletely understood. Under physiological conditions, B7-H3 protein expression is generally low, but it can be induced on several immune-cell populations, including T cells, NK cells, dendritic cells (DCs), monocytes, and macrophages [19,53]. Within the TME, B7-H3 has been associated with broad immunomodulatory activity affecting both adaptive and innate immune responses [54,55].

Several studies have reported that B7-H3 can suppress T-cell-mediated immunity by reducing the activation, proliferation, and effector function of CD4-positive and CD8-positive T lymphocytes. Mechanistically, B7-H3 has been linked to reduced IL-2 production and to negative regulation of transcriptional programs involved in T-cell activation, including NFAT, AP-1, and NF-κB [56,57]. However, its effects are not uniformly inhibitory in all settings; some studies suggest that B7-H3 may also influence Th2- and Th17-associated responses, including modulation of IL-4, IL-10, and IL-17 secretion [56]. These apparently divergent findings reinforce the view that B7-H3 function depends on the cellular and microenvironmental context.

Beyond direct effects on T cells, B7-H3 has been linked to the establishment of immunosuppressive cellular networks within the TME. Indeed, B7-H3 expression has also been associated with an immunosuppressive cytokine milieu, including increased IL-10 and TGF-β1 production, and with impaired activity of antigen-presenting cells such as DCs [19,54]. It has been associated with regulatory T-cell accumulation, although the strength and functional meaning of this association vary among tumor types [58]. B7-H3 has also been implicated in the recruitment and polarization of TAMs toward pro-tumorigenic phenotypes, including through mechanisms involving the CCL2–CCR2 axis, and in the expansion or activity of myeloid-derived suppressor cells [55,59]. In addition, B7-H3 has been reported to impair NK-cell cytotoxicity, thereby reducing innate immune-mediated recognition and elimination of malignant cells [19,54].

Overall, B7-H3 should not be assigned to a single universal immunological mechanism. Rather, it appears to contribute to immune escape through multiple, incompletely resolved pathways that converge on reduced antitumor immunity [55]. This distinction is particularly important in OSA, where immune failure is unlikely to depend on one checkpoint pathway alone, but rather on the combined effects of T-cell exclusion or dysfunction, myeloid dominance, matrix barriers, hypoxia, and metabolic stress. Importantly, several evidence linking B7-H3 to immune suppression has been generated in tumor types other than OSA, therefore, whether these mechanisms operate with similar magnitude and through the same cellular interactions in OSA need to be deeply assessed.

### 3.2. Non-Immune Functions of B7-H3: Tumor-Intrinsic and Microenvironmental Effects

In addition to its immunoregulatory activity, B7-H3 has important non-immune tumor-promoting functions. Across cancer models, B7-H3 has been associated with increased proliferation, survival, invasion, epithelial–mesenchymal transition (EMT)-like programs, stemness, resistance to apoptosis, and reduced sensitivity to chemotherapy or radiotherapy [52,60]. These functions suggest that B7-H3 may act not only as an immune-regulatory molecule, but also as a broader mediator of tumor aggressiveness.

B7-H3 expression has been linked to microenvironmental reprogramming by promoting tumor vascularization and endothelial-cell-associated programs, supporting the possibility that B7-H3 may influence not only malignant cells but also tumor-associated vascular compartments [52,60]. This is particularly relevant because abnormal vascularization contributes to hypoxia, impaired drug delivery, and immune dysfunction within the TME.

Emerging evidence also implicates B7-H3 in cancer-cell metabolic reprogramming. Several studies have shown that B7-H3 can promote glycolytic metabolism, increasing glucose utilization and lactate production, while its inhibition or silencing reduces glycolytic activity in multiple tumor models [52,61]. B7-H3 has also been associated with alterations in lipid metabolism, suggesting a broader role in metabolic plasticity [52]. By supporting these adaptive programs, B7-H3 may help tumor cells survive under unfavorable microenvironmental conditions, including hypoxia, nutrient limitation, and therapeutic stress.

This non-immune dimension is highly relevant in OSA, where tumor-cell plasticity, ECM remodeling, metastatic dissemination, metabolic stress, and chemotherapy resistance are major clinical problems. Therefore, in the context of OSA, B7-H3 should be considered as a multifunctional tumor-associated molecule that may simultaneously participate in immune restriction, and multiple tumor-promoting processes [46,52,60,62].

## 4. B7-H3 in Osteosarcoma: Expression, Prognosis, Immune Restriction, and Invasion

**Figure 2:**
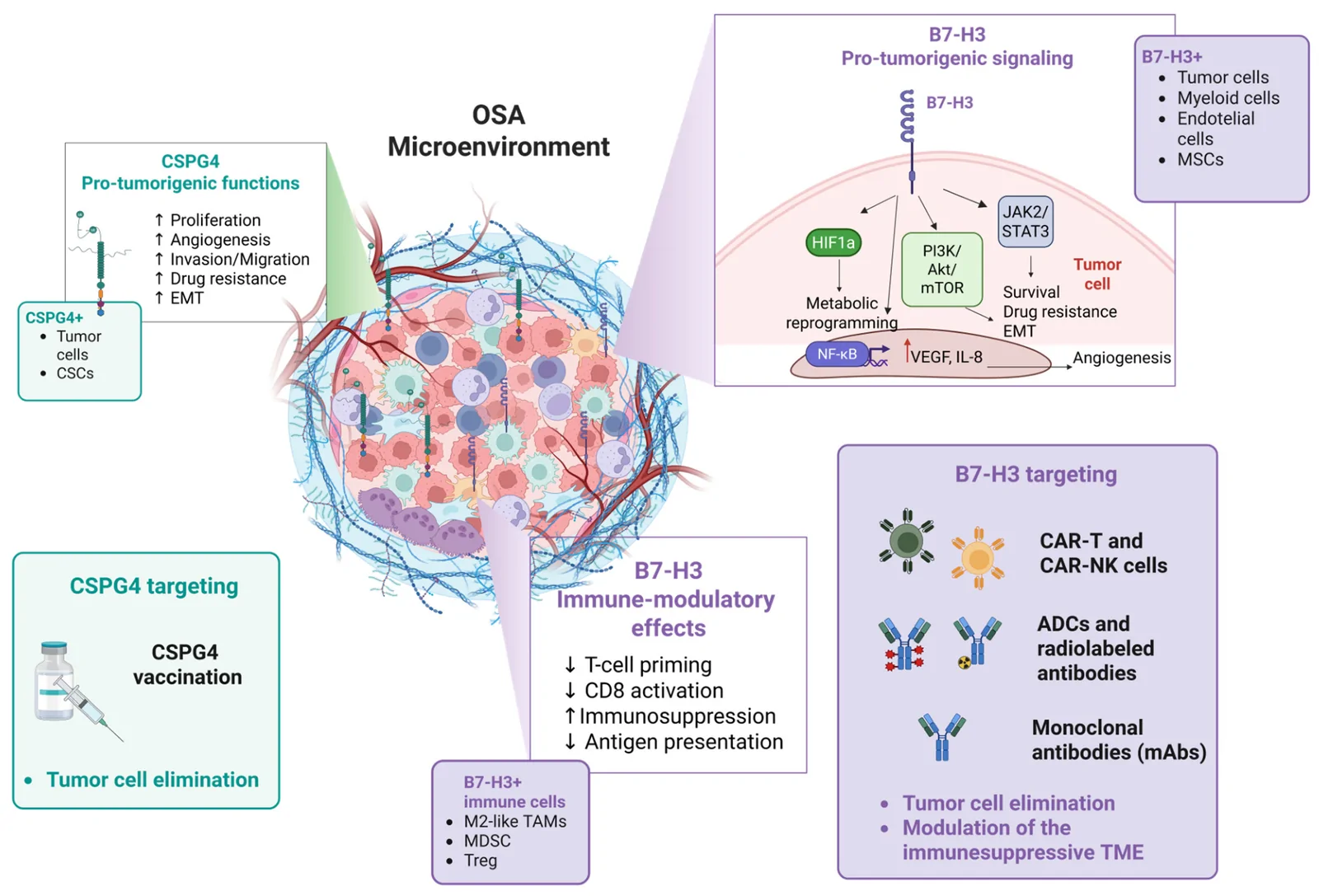
**CSPG4 and B7-H3 as complementary targets.** CSPG4 is expressed by OSA cells, including cancer stem-like populations, and is associated with tumor-promoting properties; B7-H3 is expressed by tumor and multiple microenvironmental cell populations and contributes to tumor-intrinsic and immunoregulatory processes. Their complementary activities provide a rationale for combinatorial immunotherapeutic strategies. Upward arrows indicate an increase and downward arrows indicate a reduction. Created with BioRender (https://BioRender.com/3wfkt8d), accessed on 4 October 2026.

Although many B7-H3 functions have been described across multiple tumor types [52], OSA-specific evidence remains more limited. Nevertheless, available studies suggest that B7-H3 is clinically and biologically relevant in OSA, linking tumor aggressiveness, immune infiltration, invasive behavior, and potential biomarker value [63,64,65,66,67]. Importantly, these data support the broader concept that B7-H3 should be interpreted not only as a putative immune checkpoint-like molecule but also as a multifunctional tumor-associated protein involved in both immune and non-immune aspects of OSA progression (Figure 2). In this perspective, B7-H3 may contribute to the interaction between malignant cells and the TME by associating with immune restriction, matrix remodeling, invasion, and adaptation to microenvironmental stress.

### 4.1. Tissue Expression and Biological Relevance in Osteosarcoma

The first major OSA-focused evidence showed that B7-H3 is highly expressed in OSA tissues and is associated with aggressive clinicopathological behavior. In a cohort of 61 primary OSA samples, B7-H3 expression was detected in 91.8% (56/61) lesions and was significantly higher than in adjacent normal tissues, osteochondroma, and bone fibrous dysplasia [63]. High B7-H3 expression was associated with shorter survival, shorter recurrence time, more advanced disease stage, pulmonary metastasis, and reduced CD8-positive T-cell infiltration. Functionally, B7-H3 increased OSA-cell invasion in vitro, at least in part through matrix metalloproteinase-2 (MMP-2) upregulation [63].

These findings are highly relevant because they connect B7-H3 with three key aspects of OSA biology: immune restriction, invasion, and metastasis. The association with reduced CD8-positive T-cell infiltration suggests that B7-H3-high tumors may contribute to, or at least mark, a less immune-permissive phenotype. The MMP-2 data link B7-H3 to ECM remodeling and invasive behavior [63], whereas the association with pulmonary metastasis is particularly important because lung dissemination remains the dominant cause of OSA mortality [68]. Thus, the OSA-specific evidence is consistent with the broader view of B7-H3 as a molecule positioned at the interface between immune escape, tumor-cell invasion, and metastatic competence [52,63].

At the same time, these observations should be interpreted cautiously. The available OSA data support an association between B7-H3 expression, reduced T-cell infiltration, and aggressive disease features, but they do not fully establish whether B7-H3 directly drives immune exclusion or instead reflects a broader tumor-promoting microenvironment. This distinction is important because B7-H3 may act through multiple interconnected mechanisms involving tumor cells, immune cells, stromal components, soluble mediators, and ECM remodeling. Functional studies combining B7-H3 perturbation with spatially resolved immune profiling will be required to determine whether B7-H3 is a driver of immune restriction or a marker of a broader immunosuppressive tumor state.

### 4.2. Regulatory Mechanisms and Biomarker Potential

Post-transcriptional regulation of B7-H3 may also be relevant in OSA. For example, miR-124 has been reported to directly target B7-H3; restoration of miR-124 reduced B7-H3 expression and inhibited OSA-cell growth and invasion, whereas B7-H3 re-expression counteracted these effects [64]. Other non-coding RNA mechanisms have also been explored, including hsa_circ0021347, which was identified as a B7-H3-regulated circRNA associated with malignant features of OSA cells [65]. Although these observations remain largely preclinical, they suggest that B7-H3 may participate in broader regulatory circuits controlling tumor-cell behavior rather than acting only as an isolated surface marker.

B7-H3 may also have biomarker relevance in OSA. Serum soluble B7-H3 has been investigated as a circulating biomarker and was reported to correlate with clinical parameters, tumor progression, and prognosis [66]. More recent data from the same research group have further explored soluble B7-H3 in relation to treatment response and clinical outcomes, suggesting a potential role of circulating B7-H3 dynamics during therapy [67].

These findings require independent validation, but they support the potential relevance of B7-H3 not only as a therapeutic target, but also as a candidate biomarker for risk stratification, disease monitoring, and patient selection.

Taken together, available evidence supports the view that B7-H3 in OSA may mark aggressive tumor cells, associate with reduced T-cell infiltration, support ECM remodeling and invasion, contribute to metastatic competence, and provide a therapeutically exploitable antigen.

## 5. Targeting B7-H3 in Osteosarcoma: From Antigen Discovery to Clinical Translation

The therapeutic appeal of B7-H3 stems from its dual identity as both a tumor-associated surface antigen and an immunoregulatory molecule within the TME. This biological profile has driven the development of multiple B7-H3-directed therapeutic strategies (Figure 2), which in OSA remain at the preclinical or early clinical stage [69,70].

Although available evidence supports B7-H3 association with immune restriction and malignant tumor behavior, the molecular mechanisms through which it influences the OSA microenvironment and therapeutic response remain only partially understood. Addressing these knowledge gaps will be essential for the rational design of next-generation B7-H3-targeted therapies, including the development of effective combination strategies capable of overcoming the OSA immunosuppressive and physically restrictive OSA TME, and for the identification of predictive biomarkers of response.

The main B7-H3-directed strategies can be broadly classified according to their dominant therapeutic mechanism. ADCs, radiolabeled antibodies, and cellular therapies such as CAR-T or CAR-NK cells mainly exploit B7-H3 as a surface antigen to selectively recognize and eliminate B7-H3-expressing tumor cells. In contrast, unconjugated monoclonal antibodies (mAbs) may act through Fc-mediated immune effector mechanisms, including antibody-dependent cellular cytotoxicity, antibody-dependent cellular phagocytosis, complement activation, and, potentially, partial modulation of B7-H3-associated immunosuppressive pathways. This distinction is particularly relevant in OSA, where therapeutic resistance is shaped not only by tumor-cell-intrinsic mechanisms but also by the interactions between malignant cells, immune populations, stromal compartments, ECM, and the bone niche.

### 5.1. B7-H3 as a Tumor-Associated Antigen: ADCs, Radioligands, and Cellular Therapies

The therapeutic interest in B7-H3 has rapidly led to the development of cytotoxic antigen-directed strategies, including ADCs, radiolabeled antibodies, and CAR-based cellular approaches. These platforms are conceptually appealing because they exploit the frequent and high expression of B7-H3 in OSA to achieve selective tumor-cell recognition and elimination.

Antibody engineering has made it possible to combine the specificity of mAbs with the cytotoxic activity of radioisotopes or chemotherapeutic payloads. Early proof-of-concept for B7-H3-directed radioimmunotherapy was provided by omburtamab, also known as 8H9, a murine anti-B7-H3 antibody subsequently conjugated to radionuclides such as iodine-131. Intravenous 131I-omburtamab demonstrated feasibility and acceptable safety in pediatric patients with B7-H3-positive malignancies of the central nervous system, although clinical experience in OSA remains extremely limited and this strategy has largely developed in other disease contexts [71,72,73,74].

More recently, the interest has moved toward ADCs, which combine the anti-B7-H3 antibody specificity with highly potent cytotoxic payloads (Table 1). Following antibody binding and internalization, intracellular release of the payload induces tumor-cell death while reducing systemic exposure compared with conventional chemotherapy. Several B7-H3-targeting ADCs, including ifinatamab deruxtecan (I-DXd) [75,76], HS-20093, YL201 [77,78], and MHB088C [79], and vobramitamab duocarmazine, have shown encouraging activity across B7-H3-positive solid tumors. In OSA, the most clinically relevant data currently involve HS-20093/GSK5764227, a B7-H3-directed ADC linked to a topoisomerase inhibitor payload (Table 1). In ARTEMIS-002 clinical trial, HS-20093 showed antitumor activity in relapsed/refractory OSA, with higher activity reported at the 12 mg/kg dose and a median progression-free survival of 8.4 months [80,81]. These data supported FDA Breakthrough Therapy Designation for GSK5764227 in adult patients with relapsed or refractory OSA after at least two prior lines of therapy (NCT05830123).

Preclinical studies further support the relevance of ADC-based B7-H3 targeting in pediatric OSA models (Table 1). The m276-SL-PBD, carrying a pyrrolobenzodiazepine dimer payload, showed potent antitumor activity across a large panel of pediatric patient-derived and cell line-derived xenograft models, including OSA, supporting B7-H3 as an effective ADC target in pediatric solid malignancies [82]. More recently, vobramitamab duocarmazine, a duocarmycin-based ADC, showed cytotoxic activity in pediatric sarcoma models, including OSA cells grown in both two-dimensional (2D) and three-dimensional (3D) culture systems, with effects on proliferative signaling, apoptosis, and in vivo tumor growth [83]. A multicenter preclinical evaluation from the Pediatric Preclinical In Vivo Testing Consortium extended the assessment of vobramitamab duocarmazine across a broad panel of pediatric solid tumor xenografts, including multiple OSA models. The study confirmed robust and reproducible antitumor activity across histotypes, providing strong translational support for the ongoing clinical development of B7-H3-directed ADCs in pediatric cancers [84].

Beyond antibody-based approaches, B7-H3-targeted cellular immunotherapies have emerged as one of the most actively explored strategies for selective elimination of B7-H3-expressing tumor cells (Table 1). CAR-T cells are engineered lymphocytes designed to recognize tumor-associated surface antigens independently of HLA restriction, thereby redirecting cytotoxic activity toward malignant cells. B7-H3 CAR-T cells have shown potent antitumor activity across pediatric solid tumor models, including orthotopic OSA ones, supporting the potential of this strategy [85]. Additional OSA-focused studies have confirmed that B7-H3 CAR-T cells can mediate tumor-cell killing in vitro and antitumor activity in vivo, including xenografts and metastatic models [86,87]. Importantly, these studies also highlight that B7-H3 CAR-T-efficacy is influenced not only by antigen expression but also by the ability of effector cells to access and persist within the tumor niche. Recent work specifically addressed this limitation by focusing on CAR-T cell trafficking in the OSA microenvironment. By integrating transcriptome and secretome profiling of OSA samples, Talbot and colleagues identified chemokine axes that could be exploited to enhance CAR-T-cell homing. Engineering B7-H3 CAR-T cells to express CXCR2 or CXCR6 improved their chemokine-directed migration toward OSA, increased tumor infiltration and effector functions, and prolonged survival in metastatic preclinical models [88]. These findings reinforce the concept that successful B7-H3-directed cellular therapy in OSA requires not only antigen recognition, but also active strategies to overcome microenvironmental barriers.

In addition to conventional B7-H3 CAR-T cells, alternative B7-H3-directed cellular platforms are beginning to be explored. B7-H3 CAR-NK cells have recently been evaluated in preclinical OSA models (Table 1), including engineered NK cells expressing a B7-H3 CAR together with a tumor-cleavable anti-PD-L1 scFv, showing enhanced cytotoxicity against OSA cells [89]. By contrast, B7-H3 CAR-γδ T cells remain an emerging platform supported by preclinical evidence in other solid tumors, such as glioblastoma, but their relevance in OSA has not yet been established [90].

Taken together, these studies establish that B7-H3 is therapeutically accessible and can support potent tumor-cell killing in OSA models. However, they also reveal a recurring limitation: therapeutic activity depends not only on antigen density but on antigen accessibility, immune-cell trafficking, persistence, and the ability to function within the hostile OSA microenvironment. Despite encouraging preclinical efficacy, the translation of antigen-depleting strategies into solid tumors remains challenging. Dense ECM, hypoxia, abnormal vasculature, myeloid suppression, antigen heterogeneity, and limited immune-cell persistence can all reduce therapeutic efficacy. Therefore, direct elimination of B7-H3-positive cells may not be sufficient and increasing the cytotoxic potency of B7-H3-directed platforms may not by itself overcome the biological barriers that limit durable responses. These limitations provide a rationale for combination strategies capable of simultaneously targeting malignant cells and modifying the tumor ecosystem.

**Table 1:** B7-H3/CD276-directed strategies evaluated in human B7-H3 expressing tumors, included in the main text.

Approach	Agent/Platform	Model/Setting	Main Findings	Evidence	Study ID	Ref.
**Direct killing of B7-H3^+^ tumor cells**						
**Radiolabeled** **mAb**	**Omburtamab;** humanized anti-B7-H3 antibody conjugated to iodine-131	Patients with central Nervous System/Leptomeningeal Neoplasms.	Improved survival compared to historical controls.	Clinical-Phase 1	NCT00089245	[71]
**ADC**	**Ifinatamab deruxtecan (I-DXd)**; humanized anti-B7-H3 IgG1 conjugated to DXd, topoisomerase I inhibitor, through a tetrapeptide-based cleavable linker.	B7-H3-expressing tumor cell lines; CDX and PDX models of several solid tumors; including OSA	Potent B7-H3-dependent cytotoxicity and tumor growth inhibition in preclinical models. In pediatric OSA PDXs, I-DXd induced maintained complete responses at 10 mg/kg.	Preclinical	PIVOT LB402	[76]
**ADC**	**Ifinatamab deruxtecan (I-DXd)**	Patients with advanced/refractory solid tumors, including sarcoma.	I-DXd was administered at 0.8–16 mg/kg IV Q3W. MTD was not reached. At ≥4.8 mg/kg, confirmed ORR was 34% (24/70; 95% CI 23–47) after median follow-up of 8.6 months. Responses occurred across multiple tumor types. Grade ≥ 3 anemia occurred in 18%; one treatment-related death from ILD was reported.	Clinical; Phase 1/2 dose-escalation	IDeate-PanTumor01/NCT04145622	[75]
**ADC**	**YL201**; human anti-B7-H3 mAb + novel topoisomerase I inhibitor using MediLink TMALIN tumor-microenvironment-activable linker-payload platform	B7-H3+ tumor cell lines; CDX and PDX models	Potent target-dependent cytotoxicity, cell-cycle arrest, apoptosis and bystander killing. Tumor regressions occurred in multiple CDX/PDX models at well-tolerated doses.	Preclinical	AACR 2023 #6304	[78]
**ADC**	**YL201**	Patients with advanced/refractory solid tumors.	Overall ORR reported at 40.8%, with activity across several tumor types.	Clinical, Phase 1/1b	NCT05434234/NCT06057922	[77]
**ADC**	**MHB088C;** humanized anti-B7-H3 mAb conjugated to a highly potent topoisomerase I inhibitor (SuperTopoi)	Recurrent/metastatic solid tumors.	In the reported ES-SCLC subgroup, MHB088C showed clinical activity; at the 2.0 mg/kg Q2W dose, the confirmed ORR was 42.4%, median PFS 5.9 months and DCR 87.9%.	Clinical, Phase 1/2	CTR20231298	[79]
**ADC**	**HS-20093 (GSK5764227);** fully human anti-B7-H3 IgG1 with a protease-cleavable linker and exatecan-derived TOP1 inhibitor SHR-9265.	Patients with relapsed/refractory OSA in ARTEMIS-002	In the updated analysis, 44 evaluable OSA patients were assessed. Most had stage IV disease and pulmonary metastases. ORR was 6.7% at 8 mg/kg and 17.2% at 12 mg/kg, with mPFS of 4.0 and 8.4 months, respectively.	Clinical– Phase 2	NCT05830123	[80,81]
**ADC**	**m276-SL-PBD;** anti-B7-H3 m276 antibody conjugated to PBD DNA-crosslinking payload.	Pediatric OSA xenograft models within a pediatric preclinical testing panel; randomized cohort and single-mouse studies.	Objective responses occurred in 4/5 OSA models. Treatment significantly prolonged EFS in all five models.	Preclinical	-	[82]
**ADC**	**Vobramitamab duocarmazine (Vobra Duo/MGC018);** humanized anti-B7-H3 carrying a duocarmycin-based DNA-alkylating payload.	In vitro OSA models: HOS, MG-63 and U-2-OS; 2D monolayers and multicellular tumor spheroids.	Dose-dependent cytotoxicity against OSA cells, associated with modulation of proliferative signaling and induction of apoptotic/cell-death pathways.	Preclinical	-	[83]
**ADC**	**Vobramitamab duocarmazine (Vobra Duo)**	Pediatric Preclinical Testing Consortium; 5 OSA xenograft models.	Objective responses in 3/5 OSA models. Four of five models showed EFS T/C > 2.0. Heterogeneous responses despite B7-H3 expression, need for predictive biomarkers.	Preclinical	-	[84]
**CAR-T**	**Second-generation B7-H3 CAR-T;** MGA271/CD276.MG-derived scFv, with CD8α hinge/TM and 4-1BB–CD3ζ signaling.	MG63.3 OSA cells; orthotopic tibial/hind-limb xenograft in NSG mice, including a metastatic setting following primary tumor amputation.	Regression/eradication of established MG63.3 tumors. In the metastatic model, 9/10 mice survived >5 months, whereas untreated controls died within ~50 days. Activity strongly dependent on B7-H3 surface density.	Preclinical	-	[85]
**CAR-T**	**Second-generation B7-H3.CD28ζ CAR-T;** B7-H3-specific scFv, CD8α hinge/TM and CD28–CD3ζ signaling.	LM7.ffLuc human OSA cells implanted orthotopically into the tibia of mice, generating spontaneous pulmonary metastases; metastatic setting evaluated following amputation.	B7-H3 CAR-T cells showed dose-dependent antitumor activity against primary OSA and pulmonary metastases, inhibited metastatic progression and produced a significant survival advantage.	Preclinical	-	[86]
**CAR-T**	**Third-generation B7-H3 CAR-T**; anti-B7-H3 scFv, CD8 leader/hinge/TM and 4-1BB + CD28 + CD3ζ signaling domains in tandem.	Human OSA cell lines HOS, U-2 OS, SW1353 and SaOS-2 for in vitro studies; PDX-OSTE0007 subcutaneously implanted into NCG mice for in vivo studies.	CAR-T cells efficiently killed all four OSA cell lines in vitro. In the OSA PDX, both low- and high-dose CAR-T treatment markedly suppressed tumor growth, with complete tumor disappearance in several mice and sustained tumor control.	Preclinical	-	[87]
**CAR-T**	**Second-generation B7-H3.CAR-T;** MGA271 scFv + CD8α hinge/TM + CD28–CD3ζ, co-expressing either CXCR2 or CXCR6.	143B OSA cells; orthotopic intratibial NSG mode and lung metastatic OSA model. PDX OS34 and LM7 were used for chemokine characterization/homing studies.	CXCR2/CXCR6 co-expression enhanced tumor homing and infiltration. In vivo, engineered B7-H3 CAR-T cells showed improved tumor localization and antitumor activity, with prolonged survival in metastatic models.	Preclinical	-	[88]
**CAR-NK**	**Genetically engineered B7-H3 CAR-NK;** B7-H3 CAR together with a tumor-cleavable anti-PD-L1 scFv	SaOS-2 and SaOS-2-LM7 human OSA models	Enhanced cytotoxicity against OSA cells. The secreted tumor-cleavable anti-PD-L1 scFv was functionally active and was proposed to simultaneously target B7-H3 on tumor cells and PD-L1-mediated immunosuppression in the TME.	Preclinical	-	[89]
**Immune effector mechanisms and potential microenvironmental modulation**						
**mAb**	**8H8_SDIE**	B7-H3-positive sarcoma cells including OSA	Enhanced NK activation, degranulation, IFN-γ secretion and tumor-cell killing	Preclinical	-	[91]
**mAb**	**Enoblituzumab (MGA271)** Humanized IgG1κ mAb	Human renal cell carcinoma xenografts	Potent antibody-dependent cellular cytotoxicity and inhibition of growth of B7-H3-expressing xenograft	Preclinical	**-**	[92]
**mAb**	**Enoblituzumab (MGA271)** Humanized IgG1κ mAb	Patients with relapsed or refractory solid tumors including OSA (*n* = 6).	No dose-limiting toxicities observed. No objective tumor response observed.	Clinical-Phase 1	NCT02982941	[93]

### 5.2. Unconjugated Anti-B7-H3 Antibodies: Immune Effector Mechanisms and Microenvironmental Modulation

Unconjugated mAbs offer a complementary perspective to ADCs and CAR-based therapies. Although they are generally less cytotoxic than payload-based or cellular platforms, their therapeutic effects may include Fc-mediated immune effector functions and, potentially, partial modulation of B7-H3-associated immunosuppressive pathways. Because of the poor knowledge of B7-H3 receptor, the anti-tumor effects of these antibodies may rely on antibody-dependent cellular cytotoxicity, antibody-dependent phagocytosis, complement activation, and indirect effects on immune-cell function within the TME, rather than as checkpoint blockers.

Several preclinical studies in non-OSA models have suggested that antibody-mediated B7-H3 targeting may enhance antitumor immunity by increasing T-cell and NK-cell activity, improving immune-cell infiltration, and reducing tumor growth [94,95,96]. However, the translation of these findings to OSA remains limited, partly because of the complexity of the OSA TME and the need for optimized human B7-H3–specific antibody platforms.

In OSA, a relevant example is provided by Hagelstein et al., who evaluated an Fc-optimized anti-B7-H3 antibody designed to enhance binding to Fc receptors on NK cells. This antibody, 8H8_SDIE, increased NK cell activation, degranulation, IFN-γ secretion, and effector-molecule release, leading to improved killing of B7-H3-positive sarcoma cells, including OSA cells [91]. These data support the idea that Fc-engineered B7-H3 antibodies may be useful in sarcoma by enhancing innate immune effector mechanisms.

A related strategy has been developed with enoblituzumab, also known as MGA271, a humanized IgG1 anti-B7-H3 mAb engineered to activate Fcγ receptors and reduce binding to inhibitory Fc receptors. Preclinical studies showed that enoblituzumab mediate potent antibody-dependent cellular cytotoxicity and inhibits growth of B7-H3-expressing xenograft [92]. Early-phase clinical studies in pediatric patients with solid tumors, including OSA, supported the feasibility and acceptable safety of this approach [93]. Nevertheless, clinical responses to single agent have generally been limited, possibly reflecting high tumor burden, prior therapies, limited immune effector function, and the poorly infiltrated or immunosuppressive nature of many solid tumor microenvironments.

Collectively, unconjugated anti-B7-H3 mAbs have received comparatively less attention than highly cytotoxic platforms such as ADCs or CAR-based therapies, but they remain conceptually important. By engaging immune effector mechanisms and potentially modulating B7-H3-associated immunosuppression, these antibodies may contribute to reshaping the TME. In OSA, where therapeutic failure is driven not only by intrinsic tumor resistance but also by a profoundly restrictive microenvironment, this dual mechanism may be particularly relevant. Rather than functioning solely as antitumor agents, unconjugated B7-H3 antibodies could serve as immunological modulators capable of creating a more permissive setting for complementary antigen-directed strategies.

Overall, B7-H3-directed therapies illustrate how a molecule involved in both tumor biology and immune regulation can be exploited therapeutically in OSA. ADCs and CAR-based platforms mainly use B7-H3 as a surface antigen for selective tumor-cell elimination, whereas unconjugated antibodies may additionally engage immune effector mechanisms and partially influence the immune landscape. However, none of these strategies is likely to fully overcome the complexity of the OSA microenvironment as a single agent, overall restricting therapeutic efficacy.

Together, these considerations support the development of rational combination strategies and provide a logical transition to antigen-directed approaches, such as those targeting CSPG4, which may complement B7-H3-directed therapy by enhancing tumor-specific immune recognition, providing a more permissive microenvironment that currently represents a relevant challenge for OSA management.

## 6. CSPG4 in Osteosarcoma: A Complementary Target for Antigen-Directed Immunotherapy

CSPG4 is a transmembrane proteoglycan involved in cell adhesion, migration, proliferation, ECM interactions, and intracellular signaling. It has been investigated as a tumor-associated antigen and therapeutic target in several malignancies, including melanoma, glioblastoma, triple-negative breast cancer, and sarcomas. In OSA, CSPG4 is of interest because it is expressed by tumor cells and has been linked to biological processes associated with tumor aggressiveness (Figure 2) [21,97].

Previous work from our group identified CSPG4 as a promising translational target in both human and canine OSA. CSPG4 expression was detected in OSA samples and cell lines, including stem-like osteosphere models, and was associated with shorter patients’ survival. Functionally, CSPG4 immune targeting affected OSA cell proliferation, migration, osteosphere generation, and response to doxorubicin, supporting its relevance as both a biological mediator and a therapeutically actionable antigen [22].

Considering the rarity of the disease and the paucity of fully reliable preclinical models that fully mimic the complexity of OSA, the comparative oncology dimension is particularly relevant in this context. OSA is more common in dogs than in humans, while sharing several histopathological, biological and clinical features. Because dogs develop tumors in the setting of an intact immune system, spontaneous canine OSA represents a valuable translational model for evaluating immunotherapeutic strategies and tumor–microenvironment interactions [98], even considering the near absence of murine immune competent systems.

The development of a chimeric human/dog CSPG4 DNA vaccine, hereafter referred to as HuDo-CSPG4, able to break immune tolerance against a self-non-mutated antigen, further supported the feasibility of CSPG4-directed immune targeting in comparative preclinical models of OSA. Indeed, the vaccine induced anti-CSPG4 immune responses with therapeutic potential in canine patients with spontaneous OSA. Moreover, the adoptive transfer experiments in human OSA-bearing mice supported the contribution of vaccine-induced immune components to antitumor activity also in a human setting [23]. These findings support the feasibility of CSPG4-directed immune targeting and provide a relevant example of an alternative immunotherapy in OSA.

However, the activity of targeted immunotherapy is likely to depend not only on the expression of the antigen, but also on the immune contexture of the TME. In this regard, even though HuDo-CSPG4-induced immunity significantly hampered human CSPG4-positive OSA growth, some mice showed slowed, but continuous, tumor growth. The observation that larger, less responsive OSA lesions were associated with increased B7-H3 expression and reduced CD3-positive T-cell infiltration is of particular interest [23]. This inverse relationship between B7-H3 expression and T-cell infiltration suggests that B7-H3 may mark OSA lesions with a less immune-permissive microenvironment. Nevertheless, this finding should be interpreted cautiously, as it is correlative and does not establish whether B7-H3 directly contributes to T-cell exclusion or instead reflects other features of tumor progression, such as stromal remodeling, hypoxia, myeloid suppression, or increased tumor burden.

Thus, CSPG4-directed studies provide a relevant example of a potentially effective antigen-directed immunotherapy in OSA (Figure 2) and highlight an important concept: target-antigen expression alone may not be sufficient to ensure therapeutic efficacy if the TME remains poorly permissive to immune-cell infiltration and function. This consideration provides the rationale for discussing CSPG4-directed immune targeting in relation to B7-H3.

## 7. CSPG4 and B7-H3 as Complementary Targets: Rationale for Combination Therapy

Based on the above considerations, a rational combination strategy in OSA should address two distinct but interconnected requirements: the generation of tumor-directed immune responses and the overcoming of local microenvironmental barriers that limit their efficacy. CSPG4-directed approaches mainly address the first requirement by providing an OSA-associated antigen suitable for immune recognition and vaccine-based targeting. B7-H3-directed approaches may address the second, either by directly targeting B7-H3-positive tumor or stromal compartments or by interfering with pathways associated with immune restriction and evasion.

A major limitation of antigen-directed immunotherapy is that target expression alone does not guarantee therapeutic efficacy. Even when a tumor-associated antigen such as CSPG4 is expressed and immune responses can be induced, effector cells must still reach the tumor site, persist within the local microenvironment, and retain functional activity. In OSA, these requirements may be particularly difficult to achieve because of the combined effects of immune exclusion, suppressive myeloid populations, dense extracellular matrix, abnormal vasculature, hypoxia, and metabolic stress.

Several therapeutic scenarios can be envisioned. First, CSPG4-directed vaccination could be used to prime or expand antigen-specific immune responses, with an anti-tumor benefit, while B7-H3-targeted agents could be used to eliminate B7-H3-positive tumor cells and modify B7-H3-positive tumor-associated compartments, enhancing the magnitude and effectiveness of vaccine-induced response by reducing immunosuppression. Second, B7-H3-directed cytotoxic platforms, such as ADCs or other antibody-based approaches, could be initially exploited to reduce tumor burden and potentially improve the conditions for immune-mediated control, thereby creating a more permissive environment for subsequent antigen-directed vaccination. Third, B7-H3-targeted cellular therapies or bispecific strategies could be explored in settings where B7-H3 expression is sufficiently high and spatially accessible, in combination with CSPG4 targeting to define a strategy capable of overcoming tumor heterogeneity and antigen escape, which represent important limitations of single-antigen approaches. Indeed, while antigen-directed immunity may preferentially eliminate tumor cells expressing the target, selective pressure could favor the emergence of antigen-low or antigen-negative variants. Dual targeting strategies could therefore exploit complementary mechanisms of action, simultaneously increasing tumor-cell recognition, limiting escape pathways, and promoting a more sustained antitumor response. The potential benefit of this combined approach has been suggested in the triple negative breast cancer setting, supporting the relevance of this strategy [99].

Importantly, the rationale for combining CSPG4 and B7-H3 targeting in OSA should be considered hypothesis-generating rather than established and several issues remain unresolved. It is not yet clear whether CSPG4 and B7-H3 are co-expressed by the same tumor-cell subsets, whether their expression varies between primary and metastatic lesions, or whether either molecule is modulated by chemotherapy or immune pressure. It also remains to be determined whether B7-H3 targeting can increase T-cell infiltration or function in OSA, and whether such effects would enhance CSPG4-directed immune responses. These questions are particularly relevant given the spatial and cellular heterogeneity of the OSA microenvironment.

Future preclinical studies should therefore evaluate CSPG4 and B7-H3 within the same experimental systems, ideally using models that preserve relevant features of the bone niche and immune microenvironment. Such studies should assess antigen expression, spatial distribution, CD3- and CD8-positive T-cell infiltration, macrophage and MDSC populations, vascular organization, matrix density, hypoxia, and metastatic burden. Comparative oncology models may be especially informative, as canine spontaneous OSA allows the evaluation of antigen-directed immunotherapy in an immune-competent host with clinically relevant tumor evolution.

Overall, CSPG4 and B7-H3 provide complementary perspectives on OSA immunotherapy (Figure 2). Their combined evaluation may help define more rational, biomarker-informed strategies aimed not simply at targeting tumor antigens, but at converting the OSA microenvironment into a setting more permissive to antitumor immunity.

## 8. Preclinical Models for Studying the Osteosarcoma Microenvironment and B7-H3/CSPG4-Targeted Therapy

The development of effective TME-directed and antigen-targeted therapies in OSA requires preclinical models that reproduce not only tumor-cell growth but also the biological context in which therapeutic resistance occurs. This is particularly important for strategies targeting B7-H3 and CSPG4. For B7-H3, relevant questions include antigen expression, spatial distribution, tumor versus stromal or vascular localization, immune-cell infiltration, and the relationship with invasive or immune-restricted niches. For CSPG4, model selection is equally important because antigen-directed immune responses require target expression but also depend on whether immune effectors can access and function within the TME. Therefore, no single model can fully recapitulate OSA biology; rather, different models should be selected according to the specific biological or therapeutic question being addressed.

### 8.1. Static and Dynamic 3D In Vitro Models: From Spheroids to Osteosarcoma-on-Chip Platforms

Conventional 2D OSA cell-line models remain useful for mechanistic studies, target validation, and initial drug screening. They allow controlled manipulation of B7-H3 or CSPG4 expression and can be used to assess proliferation, migration, invasion, target-dependent cytotoxicity, antibody binding, CAR-T-cell recognition, or sensitivity to ADC. However, these models poorly reproduce the spatial, mechanical, metabolic, and cellular complexity of the OSA microenvironment. They lack the mineralized bone matrix, organized extracellular scaffold, vascular components, immune infiltrates, osteoblast–osteoclast interactions, and hypoxic gradients that influence tumor progression and therapeutic response.

3D models, including spheroids, stem cell-enriched osteospheres, organoids, and scaffold-based cultures, can partially overcome these limitations. By preserving cell–cell interactions and generating oxygen and nutrient gradients, 3D systems better reproduce aspects of tumor architecture, hypoxia, metabolic stress, and therapy resistance. Osteosphere models are relevant for studying stem-like properties and therapy-resistant cell populations and the persistence of CSPG4 expression within these aggressive cell subsets [22] supports their use as a functional model to investigate the role of CSPG4 in maintaining tumor aggressiveness and therapeutic resistance.

More advanced tissue-engineered and bone-mimetic platforms may further improve the ability to model OSA-specific microenvironmental features. These systems can incorporate extracellular matrix components, mineralized scaffolds, hydroxyapatite-containing matrices, osteoblast-lineage cells, osteoclasts, endothelial cells, stromal cells, or immune cells. Recent efforts have been made to develop bioengineered 3D OSA models cultured in dynamic bioreactor systems [100] or OSA-on-chip models that address these aspects by integrating bone-like matrix components, 3D architecture, controlled fluid perfusion, and tumor–matrix interactions to study drug response and microenvironmental regulation [101,102]. Of particular relevance for immunotherapy, an OSA-on-chip model incorporating tumor-associated macrophage components has recently been proposed to investigate intratumoral heterogeneity and macrophage reprogramming in the context of immunotherapeutic strategies [103].

Dynamic microfluidic and mesofluidic systems are especially attractive because they can introduce fluid flow, controlled nutrient and drug delivery, soluble-factor transport, and, potentially, immune-cell trafficking. These features are highly relevant for B7-H3- and CSPG4-directed therapies. For B7-H3-targeted antibodies, ADC, or cellular therapies, dynamic 3D systems could help evaluate antigen accessibility, drug diffusion, effector-cell penetration, and the impact of matrix density or flow on therapeutic efficacy. For CSPG4-directed immune approaches, such platforms could be used to investigate whether antigen-specific effector cells can migrate into and act within a bone-like tumor matrix, and whether immune-cell access is affected by stromal organization, hypoxia, or macrophage-rich niches.

Overall, static and dynamic 3D models should not be viewed as replacements for in vivo systems, but as complementary platforms that allow specific microenvironmental variables to be controlled and interrogated. For B7-H3- and CSPG4-directed strategies, their main value may lie in testing whether target expression is accompanied by target accessibility and whether immune or antibody-based therapies can overcome the physical and stromal barriers that characterize OSA.

### 8.2. In Vivo Models: Anatomical Site, Immune Competence, and Metastatic Behavior

In vivo models remain essential for evaluating tumor growth, metastatic dissemination, drug delivery, immune-cell trafficking, and treatment toxicity. Subcutaneous xenografts are technically simple and reproducible, but they have important limitations for OSA research because they place tumor cells in a non-bone microenvironment and usually require immunodeficient hosts. As a result, they are poorly suited to evaluate the contribution of the bone niche or the immune system to therapeutic response.

Orthotopic and paratibial models provide a more relevant anatomical context. By allowing tumor growth in or near bone, these models better reproduce local invasion, osteolytic and osteoblastic remodeling, tumor–matrix interactions, and spontaneous metastatic dissemination, particularly to the lung. They are therefore more appropriate for testing therapies whose efficacy may depend on local tissue architecture, matrix barriers, vascularization, and metastatic progression. In the context of B7-H3-directed cellular therapy, orthotopic models have also been used to evaluate CAR-T-cell trafficking to tumor sites and activity against local and metastatic OSA [86].

Patient-derived xenografts and patient-derived orthotopic xenografts preserve aspects of interpatient heterogeneity, histology, genomic complexity, and drug sensitivity. They are valuable for testing targeted agents and for evaluating antigen expression across different patient-derived tumors. However, because they are generally established in immunodeficient mice, their use for immunotherapy is limited unless combined with humanized immune systems or ex vivo immune-cell approaches. This limitation is particularly relevant for CSPG4 vaccination and B7-H3-directed immunomodulation, where immune competence is central to the therapeutic mechanism. Patient-derived and bone sarcoma xenograft models remain valuable tools for molecular and therapeutic studies, but their immune limitations should be considered when interpreting immunotherapy results [104].

The model used by Tarone et al. [23] provides an instructive example of how this limitation can be partially addressed in antigen-directed immunotherapy studies. Human OSA cells were implanted in immunodeficient NSG mice (NOD-scid IL2Rγnull), a severely immunodeficient strain lacking mature T cells, B cells, and functional NK cells, which permits engraftment of human tumor cells. In parallel, HuDo-CSPG4 vaccination was performed in immune-competent C57BL/6 mice transgenic for HLA-A2, an MHC context matched with the human OSA cell line used in the xenograft model. Vaccine-induced immune components, including CD8-positive T cells and sera, were then transferred into human OSA-bearing NSG mice, where they delayed tumor growth and metastasis development [23]. This strategy illustrates a useful compromise: the xenograft host permits human tumor growth, while the vaccination phase preserves immune competence and allows the generation of antigen-specific effector components in a human-relevant HLA context.

At the same time, this approach also highlights the limitations that remain when modeling immunotherapy in OSA. Because the tumor-bearing host is immunodeficient, the model cannot fully reproduce endogenous immune priming, long-term immune memory, myeloid suppression, stromal–immune crosstalk, or the complete bone-associated immune microenvironment. Therefore, such models are best interpreted as translational systems to test whether vaccine-induced immune components can recognize and control human OSA targets, rather than as complete models of the OSA immune microenvironment.

Syngeneic murine models offer the advantage of an intact immune system and are therefore important for studying macrophages, MDSCs, T cells, immune checkpoints, vaccine responses, and combination immunotherapy. They may be useful to investigate whether microenvironmental modulation can enhance antigen-directed immune responses. However, species differences in tumor biology, immune regulation, antigen structure, and B7-H3 isoform biology must be considered. For example, murine B7-H3 differs structurally from the predominant human 4Ig-B7-H3 isoform, which may affect the interpretation of B7-H3-targeted strategies. Therefore, syngeneic models are informative for immune mechanisms, but not always directly predictive for human antigen-targeted therapies.

Humanized mouse models may help bridge this gap by allowing evaluation of human immune-cell interactions with human tumor cells. However, these models remain technically complex and incompletely reproduce the full human immune system, bone microenvironment, and myeloid compartment. They may be useful for specific questions related to human CAR-T cells, bispecific antibodies, or antibody-based approaches, but their limitations should be acknowledged when interpreting results. For example, B7-H3-targeted CAR-T cells have shown antitumor activity against OSA in vitro and in vivo, including in patient-derived xenograft settings, but the predictive value of these systems for clinical immune function remains constrained by the model context [87].

### 8.3. Comparative Oncology: From Target Discovery to Translational Immunotherapy

Comparative oncology provides a particularly relevant translational opportunity for OSA. Human OSA is rare, biologically heterogeneous, and clinically difficult to treat, which limits the speed and scale of therapeutic development. Spontaneous canine OSA, by contrast, occurs more frequently and shares several clinical and biological features with human disease, including aggressive local behavior, metastatic propensity, and resistance to the same conventional therapies, which include surgery and chemotherapy. These characteristics make canine OSA a valuable intermediate model between murine systems and human trials, particularly for immunotherapeutic strategies and tumor–microenvironment studies [5].

The CSPG4 studies from our group illustrate the value of this comparative approach. In the paper published by Riccardo et al. we identified CSPG4 as a promising target in both human and canine OSA, showing CSPG4 overexpression in human high-grade OSA and canine OSA samples, association with shorter survival, and functional effects of CSPG4 immune targeting on proliferation, migration, osteosphere generation, and response to doxorubicin [22]. This work established a biologically and translationally grounded rationale for pursuing CSPG4 as an OSA-associated antigen across species.

The subsequent development of the HuDo-CSPG4 chimeric human/dog DNA vaccine further extended this strategy. In the work published by Tarone et al. we evaluated the HuDo-CSPG4 vaccine in comparative preclinical models, including not only human OSA xenograft mouse models, but also canine patients with spontaneous OSA. The therapeutic potential of the vaccine induced anti-CSPG4 immune responses in canine patients was strongly envisaged [23], providing a concrete example of how comparative oncology can support the translational development of antigen-directed immunotherapy.

From a translational perspective of combinatorial approaches development, DNA vaccination platform itself offers several advantages. Plasmid DNA vaccines are relatively simple to manufacture, and modify, and are versatile, allowing rapid design of combination strategies. Their immunogenicity can be substantially enhanced by in vivo electroporation, which improves plasmid uptake and antigen expression. This delivery platform has already been extensively investigated in veterinary oncology, including in studies targeting CSPG4 in canine melanoma and OSA patients [23,105,106,107,108,109,110,111,112]. In all these studies, antigen-directed DNA vaccination in association to electroporation demonstrated a favorable safety profile together with the induction of robust antigen-specific immune responses. Given the growing clinical application of electroporation-based DNA delivery in humans, these findings further acquire a high translational meaning.

All these aspects, including the delivery method, immune competence, antigen conservation across species, and spontaneous tumor evolution influence the translational value of vaccine-based approaches [5,113,114].

### 8.4. Model Selection for B7-H3/CSPG4-Directed Strategies

The comparative framework is particularly relevant for the present review because B7-H3- and CSPG4-directed strategies depend on more than target expression alone. ADCs require models that preserve antigen density, internalization, drug sensitivity, and tumor heterogeneity. CAR-T cells, bispecific antibodies, and vaccines require models that allow assessment of immune-cell trafficking, persistence, exhaustion, and activity within a restrictive microenvironment. Strategies intended to modulate the TME require models that include relevant stromal, vascular, myeloid, and matrix components.

For B7-H3-directed cellular therapy, recent preclinical studies highlight the importance of modeling immune-cell trafficking and homing. Orthotopic OSA models have been used to demonstrate B7-H3 CAR-T-cell trafficking to tumor sites and activity against spontaneous metastases [86]. Additional work has shown that redirecting B7-H3 CAR-T cells toward chemokines expressed by OSA can improve homing and antitumor activity, emphasizing that antigen recognition alone may not be sufficient if effector cells cannot efficiently reach tumor sites [88]. These observations are conceptually aligned with the broader theme of this review: successful antigen-directed therapy must overcome both target-recognition and microenvironmental-access barriers.

For CSPG4-directed vaccination, immune-competent and comparative models are particularly important because the therapeutic mechanism depends on the induction, maintenance, and effector function of antigen-specific immune responses. The use of spontaneous canine OSA allows evaluation of vaccine-induced immunity in the presence of naturally evolving tumor heterogeneity, stromal remodeling, immune suppression, metastatic progression, and prior or concurrent standard-of-care treatment. This makes comparative oncology especially suitable for testing whether antigen-directed immunotherapy can function within a clinically relevant and immunologically intact tumor microenvironment [5,23].

Future studies should therefore use integrated model systems capable of capturing the major biological determinants of therapeutic response, including antigen expression and accessibility, immune-cell infiltration, macrophage and MDSC populations, vascular organization, matrix density, hypoxia, and metastatic behavior. Model selection should be guided by the specific question being addressed: dynamic 3D systems may be particularly useful for dissecting drug penetration and immune-cell trafficking, whereas immune-competent, humanized, or comparative oncology models are required to investigate immune activation, suppression, and treatment-induced remodeling of the TME. Combining complementary experimental systems may therefore provide a more informative framework than relying on a single model to reproduce the full complexity of OSA.

Overall, preclinical models should be viewed not only as platforms for measuring tumor growth inhibition, but as tools to test whether a therapeutic strategy can overcome the biological barriers that define OSA. The comparative-oncology work on CSPG4 demonstrates how human and canine models can be combined to move from target identification to immune-based translational development. This same logic may be useful for future studies evaluating B7-H3/CSPG4-directed combinations or other strategies aimed at converting the OSA microenvironment into a setting more permissive to antitumor immunity. In this perspective, model selection becomes part of therapeutic design, not merely a technical step preceding clinical translation.

## 9. Toward Biomarker-Guided and Microenvironment-Informed Immunotherapy: Unresolved Questions and Future Directions

Despite significant advances in the molecular characterization of OSA, the identification of biomarkers capable of predicting response to therapy and guiding patient stratification remains a critical challenge. Current tissue-based biomarkers primarily rely on immunohistochemistry or bulk transcriptomic analyses to assess the expression of proteins and genes associated with tumor aggressiveness, metastatic propensity, and response to therapy. Few tissue biomarkers have been related to clinical outcome in OSA, including immune-related molecules such as B7-H3 [63,69] and PD-L1 [115,116], angiogenic mediators such as VEGF [117], proliferation markers including Ki-67 [118], and genetic alterations affecting TP53 [119,120]. Additional tumor-associated antigens, including CSPG4 [22] and ezrin [121,122], have also been associated with poor prognosis and metastatic propensity; nevertheless, histological response to neoadjuvant chemotherapy remains the most widely adopted prognostic indicator in clinical practice [123]. Circulating biomarkers, including circulating tumor DNA [124], microRNAs [125], extracellular vesicles [126,127,128], and soluble proteins [67], have also emerged as promising minimally invasive tools for monitoring disease burden and early detection of recurrence. However, despite encouraging results, none of these biomarkers has yet demonstrated sufficient reproducibility or predictive performance for routine clinical implementation.

One of the principal reasons underlying this limited clinical translation is the remarkable biological heterogeneity of OSA. Conventional tissue analyses evaluate the average molecular profile of bulk tumor specimens, thereby masking the coexistence of multiple malignant, stromal, and immune cell populations with distinct biological functions. Consequently, biomarkers measured in a single biopsy may incompletely represent the entire tumor ecosystem and fail to capture the dynamic interactions and the spatial organization within the TME, that ultimately determines disease progression and therapeutic response.

Recent advances in single-cell RNA sequencing (scRNA-seq) have substantially improved our understanding of OSA biology by enabling transcriptomic profiling at single-cell resolution. Studies have generated high-resolution atlases of treatment-naïve OSA, identifying diverse malignant, stromal, and immune populations together with their transcriptional programs, differentiation trajectories, and functional states [129,130,131,132]. Beyond cellular classification, single-cell approaches have reconstructed intercellular communication networks through ligand-receptor analyses, revealing signaling pathways that regulate angiogenesis, osteoclast activation, immune suppression, and tumor progression.

Although these studies have established a valuable framework for dissecting the cellular architecture of the OSA microenvironment, the field is still in its early stages. Most published single-cell datasets include relatively small patient cohorts, reflecting both the rarity of OSA and the considerable technical challenges associated with processing bone tumors [25,129,133,134,135,136]. The mineralized nature of bone tissue, its dense ECM, and the need for extensive enzymatic and mechanical dissociation complicate the generation of viable single-cell suspensions, potentially introducing technical bias and selective loss of specific cellular populations.

Nevertheless, these studies have consistently highlighted the remarkable intra- and inter-tumoral heterogeneity of OSA, revealing previously unrecognized malignant cell states, diverse immune and stromal populations, and complex intercellular communication networks that cannot be resolved using conventional bulk transcriptomic approaches. Future efforts should focus on validating these observations in larger, multicenter cohorts and integrating the findings with functional studies and clinical outcome data. Such validation will be essential to distinguish biologically relevant cell states and to translate single-cell discoveries into clinically actionable biomarkers and therapeutic targets.

Moreover, the tissue dissociation required for scRNA-seq inevitably disrupts the spatial organization of the tumor. Emerging spatial transcriptomic and multiplex imaging technologies are now overcoming this limitation by preserving tissue architecture while simultaneously profiling gene expression, better revealing the heterogeneity and spatial compartmentalization of the TIME and providing comprehensive atlas of infiltrated immune cell types in OSA [131,137,138]. These approaches allow the identification of spatially organized cellular neighborhoods and immune niches within their native anatomical context. Importantly, these approaches can reveal not only which immune and stromal populations are present, but they can determine whether cytotoxic lymphocytes successfully infiltrate tumor nests or remain confined to the invasive margin, whether immunosuppressive macrophages and stromal populations colocalize with malignant cells, and how these spatial relationships influence metastatic progression, prognosis, and response to (immune) therapy. Such spatially resolved analyses are expected to substantially improve patient stratification and support the development of biomarker-guided therapeutic strategies.

Within this framework, B7-H3 represents a particularly attractive candidate biomarker, for which spatial characterization represents an important unmet need in OSA. Indeed, its expression extends across multiple cellular compartments within the OSA microenvironment. Future studies should determine which cellular compartments preferentially express B7-H3, whether its expression colocalizes with exhausted T cells, tumor-associated macrophages, endothelial cells or mesenchymal stromal populations, and how these spatial relationships evolve during therapy. Such information will likely provide insights that cannot be obtained through conventional bulk analyses alone. Integrating spatial biology with single-cell transcriptomics may therefore identify biomarkers predictive of response to B7-H3-directed therapies and inform the rational design of combination immunotherapeutic strategies.

Collectively, integrating single-cell and spatial multiomic technologies with functional immunological studies will be instrumental for identifying predictive biomarkers, refining patient selection, and guiding the rational design of combination immunotherapies targeting both tumor cells and the OSA microenvironment. However, the increasing complexity generated by these analyses will require computational frameworks capable of integrating heterogeneous biological information. Artificial intelligence and machine-learning approaches are emerging as powerful tools to identify multidimensional biomarker signatures, prioritize therapeutically relevant targets, and generate predictive models that link antigen expression, immune composition, spatial architecture, and clinical outcome. Rather than replacing experimental validation, these approaches provide a data-driven framework for biomarker discovery and precision immunotherapy, offering the opportunity to move beyond single-marker evaluation toward comprehensive, microenvironment-informed clinical decision making in OSA [139,140] as well as in other tumor types [141,142,143].

### Outstanding Questions

Despite these advances, several fundamental questions remain unresolved. First, it is still unclear whether B7-H3 directly contributes to immune restriction in OSA or primarily identifies and aggressive, poorly immune-permissive tumor state. Establishing causality will help determine whether B7-H3 targeting can actively remodel the TME in addition to eliminating B7-H3-positive cells.

Second, the cellular and spatial distribution of B7-H3 within OSA remains incompletely defined. Future studies should determine the relative contribution of malignant, vascular, stromal, and immune-cell compartments to B7-H3 expression and establish whether this distribution changes between primary and metastatic lesions or during treatment.

Third, the extent of intra- and intertumoral heterogeneity of therapeutically relevant antigens remains poorly characterized. In particular, it will be important to determine whether B7-H3 and CSPG4 co-expressed by the same tumor-cell populations or identify partially distinct cellular compartments, and whether their expression is modified by chemotherapy or immune pressure. These questions are particularly relevant to the development of combined or sequential targeting strategies.

Another unresolved issue is whether B7-H3-directed therapy can improve the effectiveness of antigen-specific immunity. Although B7-H3-targeted approaches can eliminate antigen-positive cells, it remains to be established whether they can also enhance T-cell recruitment, persistence, or function within OSA lesions and thereby increase the efficacy of CSPG4-directed or other antigen-specific immune responses.

Finally, the biomarkers required to guide these approaches remain undefined. B7-H3 expression alone may not be sufficient to predict therapeutic response, and future patient stratification may require integration of antigen density and localization with immune-cell composition, myeloid states, spatial organization, circulating biomarkers, and treatment-induced changes in the TME. Defining such multidimensional biomarkers will be essential for determining not only which patients should receive a given therapy, but also the most appropriate timing and combination of tumor-directed and microenvironmental-directed interventions.

Addressing these questions will require moving beyond descriptive measurements of target expression toward longitudinal, spatially resolved, and functionally validated analyses of the mechanisms and to translate microenvironment-informed therapeutic concepts into rationally designed clinical strategies.

## 10. Conclusions

OSA remains a major therapeutic challenge because it is not only genetically heterogeneous but also microenvironmentally protected. Its bone-associated TME supports tumor progression while restricting effective antitumor immunity. Suppressive myeloid cells, excluded or dysfunctional T cells, abnormal vasculature, dense mineralized matrix, hypoxia, metabolic stress, and osteoblast–osteoclast remodeling collectively create a tumor niche that favors metastatic dissemination and therapeutic resistance.

Successful immunotherapeutic approaches in OSA will likely require strategies capable of addressing not only tumor-cell intrinsic mechanisms but also the complex interactions between malignant cells and their surrounding microenvironment.

B7-H3/CD276 represents a central molecule within this landscape. Although its receptor biology remains unresolved, B7-H3 is strongly implicated in immune regulation, tumor invasion, metastasis, angiogenesis, metabolic adaptation, and treatment resistance. In OSA, its association with poor outcome and reduced T-cell infiltration supports its role as a marker and possible mediator of immune restriction, although a causal relationship remains to be established. Its cell-surface expression further makes it an attractive therapeutic target for ADCs, CAR-T cells, bispecific antibodies, and mAbs.

In parallel, the CSPG4 molecule provides a complementary antigenic axis: as an OSA-associated antigen involved in tumor aggressiveness and amenable to vaccine-based targeting, CSPG4 can be exploited to promote immune activation against tumor cells. However, antigen-specific immune priming alone may be insufficient if effector cells encounter an immune-excluded and suppressive microenvironment. Our observation that larger OSA lesions show increased B7-H3 expression together with reduced CD3-positive T-cell infiltration supports the hypothesis that antigen-directed immunity and microenvironmental regulation are interconnected determinants of immune responsiveness and that B7-H3 expression may identify an immune-restricted tumor state in which antigen-directed immune responses are potentially impaired by limited immune-cell recruitment and/or functional suppression.

Therefore, CSPG4 and B7-H3 may be considered not as alternative targets but as complementary components of a multidirectional immunotherapeutic strategy. A future therapeutic model for OSA may involve priming or redirecting immunity against tumor-associated antigens such as CSPG4 while simultaneously targeting B7-H3 to eliminate aggressive tumor compartments and relieve microenvironmental barriers to immune infiltration.

In parallel to the development of innovative therapeutic approaches, advances in scRNA-seq, spatial transcriptomics, and artificial intelligence-driven biomarker integration will be essential to identify which patients are most likely to benefit from these strategies and to define the spatial and cellular determinants of therapeutic response.

Future progress in OSA immunotherapy will also depend on the development of more physiologically relevant experimental models capable of recapitulating the complexity of the human tumor ecosystem. Conventional 2D cultures and immunodeficient xenograft models provide important mechanistic insights but frequently fail to reproduce the interactions between malignant cells, bone-associated stromal components, immune populations, extracellular matrix architecture, and therapeutic barriers that characterize the native tumor microenvironment. The implementation of advanced 3D models, including organoid-based systems, bone-mimetic matrices, and microfluidic platforms capable of modeling perfusion, drug diffusion, and immune-cell trafficking, may provide more realistic tissue-like environment and improve the predictive value of preclinical studies.

Comparative oncology represents an additional and complementary translational opportunity for OSA research. Spontaneous canine OSA shares several clinical and biological features with human disease, providing a clinically relevant intermediate model between experimental systems and human trials, allowing evaluation of novel therapeutic approaches in an intact immune system and in the context of naturally occurring tumors. Integration of advanced preclinical models with comparative oncology approaches may accelerate the identification of effective therapeutic strategies and improve the translation of immunomodulatory treatments into OSA clinical practice.

Ultimately, the future of OSA immunotherapy will likely depend on moving beyond single-marker targeting toward microenvironment-informed and biomarker-guided strategies that not only eliminate tumor cells but can reshape the OSA niche from a tumor-supportive and immune-hostile ecosystem toward one that permits durable and effective antitumor immunity. Achieving this goal will require resolving the causal role of B7-H3 in immune restriction, defining the spatial and temporal heterogeneity of therapeutically relevant antigens, and establishing biomarkers and treatment sequences capable of guiding combination strategies.

## Data Availability

No new data were created or analyzed in this study. Data sharing is not applicable to this article.

## Abbreviations

The following abbreviations are used in this manuscript:

OSA	Osteosarcoma
ICI	Immune checkpoint inhibitors
TME	Tumor microenvironment
ADC	Antibody–drug conjugates
CAR-T	Chimeric antigen receptor-T-cell
CSPG4	Chondroitin sulfate proteoglycan 4
TLR2	Toll-like receptor 2
ECM	Extracellular matrix
TAMs	Tumor-associated macrophages
MDSCs	Myeloid-derived suppressor cells
MSC	Mesenchymal stem cells
NK	Natural killer
DCs	Dendritic cells
EMT	Epithelial–mesenchymal transition
MMP-2	Matrix metalloproteinase-2
mAbs	Monoclonal antibodies
I-DXd	Ifinatamab deruxtecan
2D	Two-dimensional
3D	Three-dimensional
NSG	NOD-scid IL2Rγnull
scRNA-seq	single-cell RNA sequencing
